# The joint effect of framing and defaults on choice behavior

**DOI:** 10.1007/s00426-022-01726-3

**Published:** 2022-09-05

**Authors:** Felice Giuliani, Loreta Cannito, Gilberto Gigliotti, Angelo Rosa, Davide Pietroni, Riccardo Palumbo

**Affiliations:** 1 Center for Advanced Studies and Technologies “CAST”, University “G. d’ Annunzio” of Chieti-Pescara, Via Luigi Polacchi, 11, 66100 Chieti, Italy; 2 Department of Neuroscience, Imaging and Clinical Sciences, University “G. d’ Annunzio” of Chieti-Pescara, Chieti, Italy; 3 Department of Psychological Sciences, Health and Territory, University “G. D’Annunzio” of Chieti-Pescara, 66100 Chieti, Italy; 4 Department of Management, Finance and Technology, University “LUM”, Casamassima, Bari, Italy

## Abstract

The framing effect leads people to prefer a sure alternative over a risky one (risk aversion) when alternatives are described as potential gains compared to a context-dependent reference point. The reverse (risk propensity) happens when the same alternatives are described as potential losses. The default effect is the tendency to prefer a preselected alternative over other non-preselected given options, without facilitating nor incentivizing the choice. These two effects have mainly been studied separately. Here we provided novel empirical evidence of additive effects due to the application of both framing and default within the same decision problem in a large sample size (*N* = 960). In the baseline condition, where no default was provided, we measured the proportion of risky choices in life-or-death and financial decisions both presented in terms of potential gains or losses following the structure of the Asian disease problem. In the sure default condition, the same layout was proposed with a flag on the sure option, whereas in the risky default condition, the flag was on the risky option. In both default conditions, we asked participants whether they wanted to change the preselected option. Overall, the comparison between these conditions revealed three distinct main effects: (i) a classic framing effect, (ii) a larger risk propensity in the life-or-death scenario than in the financial one, and (iii) a larger default effect when the flag was on the risky, rather than on the sure, option. Therefore, we conclude that default options can enhance risk propensity. Finally, individual beliefs about the source of the default significantly moderated the strength of the effect. Underlying mechanisms and practical implications are discussed considering prominent theories in this field.

**Supplementary Information:**

The online version contains supplementary material available at 10.1007/s00426-022-01726-3.

## Introduction

Since the pioneering work of Herbert Simon (Simon, 1955), in the early years of bounded rationality, a paradigm shift in the study of decision-making has been taking us closer to the understanding of real decision-makers, rather than of abstract hypothesized rational beings. In this paper, we foster this type of understanding, using a descriptive approach, which tries to explain how individuals evaluate alternatives and make decisions, possibly specifying the underlying cognitive mechanisms (for further details on this topic see for instance: Wheeler, 2020; Baron, 2012).

Our empirical investigation builds on previous findings of classic behavioral effects in this field, considering two distinct but connected phenomena. The first one is the starting point of our empirical analysis. It consists of the systematic tendency to prefer a sure option over a fairly risky one when a decision problem is framed in terms of potential gains. This tendency reverses when the same decision task is presented in terms of potential losses. This phenomenon is known as the framing effect (Tversky & Kahneman, 1981).

The second effect under examination is considered rather effective in directing individuals’ choices, but not yet well understood in terms of underlying psychological mechanisms (Dinner et al., 2011; Jachimowicz et al., 2019). Behaviorally, it is the tendency to stick with a preselected option, which leads to a shift in preferences at the expense of alternative options. This phenomenon is known as the default effect, which can be associated with, but not limited to, the status quo bias, that is the behavioral tendency to keep the current situation unchanged when facing a decision problem (Samuelson & Zeckhauser, 1988). As we will illustrate in the following sections, both effects seem to be based on reference-dependent judgments, but rather interestingly, we do not know what ought to be observed when a decision problem is both framed as a potential gain/loss and, simultaneously, one of the available options is presented as a preselected choice. In other words, currently, it is difficult to predict which one of the two effects would set the reference, if any, and how this would happen.

We believe that this problem may be of extreme relevance to (i) clarify which one of the two effects has the largest impact on choice behavior, (ii) enhancing our understanding of underlying psychological mechanisms, and therefore (iii) providing more detailed instructions to those who would apply these principles in practical contexts. To the best of our knowledge, no research has been explicitly devoted to answering this research question in decision-making under risk, except for one single preliminary study that has begun to uncover some interesting mechanisms regarding the effect of the default option in gambling decisions (Costa-Gomes & Gerasimou, 2020). For the sake of completeness, it should be mentioned that studies combining framing and default effects do exist, although they do not measure the proportion of risky choices, but only the acceptance rate of an option that is positively framed compared to an option that is negatively framed (that is the negated form of the positive statement; Johnson et al., 2002).

### Framing effect

In choice behavior, the simplest way to define the framing effect is a difference in preferences that occurs when individuals are confronted with two different, but logically equivalent descriptions of the same decision problem (Wallin et al., 2016).

Considering the following example integrally reported from Tversky and Kahneman (1981):

*“Imagine that the United States is preparing to face an Asian disease that, given its exceptional severity, could cause the death of 600 people. Two alternative intervention programs are proposed to deal with this event. Assume that the exact scientific estimate of the consequences of the two programs is:”*

*Gain frame*

*“If Program A is adopted, 200 people will be saved.*

*If Program B is adopted, there is 1/3 probability that 600 people will be saved,* and *2/3 probability that no one will be saved.*

*Which one of the two programs would you favor?”*

*Loss frame*

*“If Program C is adopted, 400 people will die.*

*If Program D is adopted, there is 1/3 probability that nobody will die,* and *2/3 probability that 600 people will die.”*

*Which one of the two programs would you favor?”*

In the original experiment (Tversky & Kahneman, 1981), the two versions of the problem were presented to two different experimental groups. Participant’s preferences were strongly polarized towards the sure option A in the gain frame (approximately 72%), and towards the risky option D in the loss frame (approximately 78%). When presented together, it is easy to notice that the two descriptions are the mirrored version of one another. Specifically, 200 people saved (A) implies that 400 people die (C), and the same applies to B (600 saved/no people saved) and D (no people die/600 people die). Therefore, the two versions are differently described but logically equivalent.

The difference in preferences, originally described by Tversky and Kahneman (1981), violates the principle of invariance, which states that people’s choices should remain constant even when the surface description of a decision problem changes. Over the years, this phenomenon has attracted a considerable amount of attention since it challenges some core assumptions of the dominant normative decision theory.

This phenomenon has been originally explained by Prospect Theory (PT; Kahneman & Tversky, 1979; Tversky & Kahneman, 1981). In PT, the different options are called prospects, and the way the subjective utility of the outcomes is evaluated is similar to previous formal models, such as the Expected Utility Theory (EUT). In short, the psychological value of a prospect is the result of the utility of the outcomes multiplied by their probabilities. Importantly, both distributions of outcomes and probabilities are supposed to be represented non-linearly in the human mind. Specifically, the subjective pleasure or utility declines as the outcomes increase (Bernoulli, 1954; Kahneman & Deaton, 2010; Kahneman & Tversky, 1979). In psychology, this type of nonlinearity has been found in the way individuals rate monetary values on an affective basis (Giuliani et al., 2021; Manippa et al., 2021), and estimate prices (Giuliani et al., 2017; Raposo et al., 2018). For this reason, in the gain domain, a sure prospect of 200 may have more subjective utility than a risky prospect of 600 with a probability of 1/3, even though, mathematically, 1/3 of 600 is in fact 200.

Since in PT, the utility function is supposed to reverse in the loss domain, thus assuming negative values, a given prospect that has a higher positive value in the gain domain will have a higher negative value in the loss domain, thus leading people to avoid it. Hence, the S-shaped value function postulated by PT describes the tendency to be *risk averse* with potential gains and *risk seeker* with potential losses of the same amount.

It is worth noticing that in the above-reported example, the outcomes are potential losses, but they can appear to be potential gains when compared to a reference point determined by the words used to describe the outcomes. The use of the word “saved” (gain frame) implies that the reference is “600 people dead”, thus prospects fall in the gain domain and choices lean towards the safe option. In contrast, the use of the word “die” (loss frame) implies that the reference is “zero people dead”, thus prospects fall in the loss domain and choices lean towards the risky option.

Nonetheless, there are some alternative and conflicting interpretations that contribute to improving our understating of this type of framing effect and its underlying mechanisms. PT has been classified into the category of “value-first decision-making”, which has been questioned by “comparison-based decision-making without value computation”, a class of theories that rejects the core assumptions of EUT and PT (Vlaev et al., 2011). For instance, within the family of fast and frugal heuristics (Drechsler et al., 2014; Gigerenzer, 2004), the Priority heuristic (Brandstätter et al., 2006) postulates that, when facing decision problems such as a binary choice between two alternative gambles, we search for pieces of information in a certain order (lexicographic), determining a hierarchy of reasons and stopping as soon as one reason reaches the aspiration level (“good enough”), otherwise we go ahead evaluating the next reason. Importantly, the aspiration level is based on a comparative judgment between the options and the first judgment focuses on the outcomes, without considering probabilities. In short, if the difference between minimum gains exceeds a certain portion of the maximum gain, the option with the *higher minimum gain* is chosen. The same applies to losses, with the only difference that the more attractive option has the *lower minimum loss*. Only if this condition is not met, probabilities of minimum gains/losses are considered.

Although this heuristic accounts for the *reflection effect*, which is observed when real gains and losses are involved, it illustrates that value functions are not always necessary to explain choice behavior under risk and uncertainty. In the same vein, along the line of non-utility/value-based frameworks, the Fuzzy-trace theory (Brainerd & Reyna, 1990) and the Evaluative Polarity account (Wallin et al., 2016) have been focused on linguistic and affective mechanisms as determinants of the framing effect.

Wallin et al. (2016) have demonstrated that the difference between the perceived pleasantness of the options predicts the choice and that this relative comparison is only based upon the words used to describe the options, regardless of the positive or negative formulation of the problem. This account assumes that no domains are created based on reference points, and no mental operations on probabilities and outcomes are performed. Kühberger and Tanner (2010) argued that fuzzy-trace theory would provide a similar account, assuming a direct comparison between options, in which, in the gain frame, the sure gain is preferred over the risky gain because the latter mentions the possibility that someone will not be saved, whereas the former does not (it only mentions that 200 people will be saved). In contrast, in the loss frame, the risky loss is preferred over the sure loss because the former mentions that someone will not die, whereas the latter does not (it only mentions that 400 people will die).

Analyzing these models, it becomes clear that they are not based on any independent assignment of value to the prospects before their comparison, but instead they are centered on the relative comparison between a simplified representation of the options.

The framing effect can occur in different contexts, demonstrating the robustness and pervasiveness of such phenomenon. For instance, in a classic study, McNeil et al. (1982) found that patients with lung cancer found the risk of surgery to be acceptable when the rate of success was presented in terms of probability of living than in terms of the probability of dying. More recent research, analyzing real medical consultations, has confirmed that this bias is present in the way physicians propose either active surveillance or treatment through surgery or radiation to their cancer patients. In turn, the use of cancer survival or cancer mortality-related words seems to ultimately influence patients’ decisions (Fridman et al., 2020).

### Default effect

As stated above, the default effect is the tendency to adopt the preselected option, determining a choice bias toward the default while penalizing the alternative option. There are two paradigmatic and very straightforward examples illustrating how this phenomenon works. The most famous is likely the default effect on organ donation, reported by Johnson and Goldstein (2003). They have demonstrated how the status quo of not being an organ donor, who can choose to become one (opt-in), leads to a lower rate of organ donations. In contrast, the status quo of being an organ donor, who can choose to withdraw from that status (opt-out), leads to a greater rate of organ donations.

Another well-known example has been reported by Madrian and Shea (2001), demonstrating that when employees are automatically enrolled in a retirement plan (opt-out), it is 50% more likely that they will stay with that default, therefore having a retirement plan, compared to the group for which the default position was not to be enrolled in any retirement plan (opt-in). Finally, a further example can be found in Pichert and Katsikopoulos (2008) who have demonstrated how defaults can be used to foster “environmentally friendly” choices in terms of energy usage.

These three examples seem to provide a clear, simple, and effective rule that can be useful in several contexts: whenever a choice needs to be encouraged to reach a given goal, present it as the default option, namely something people need to opt out of to make a different choice. This will maximize the percentage of people who passively “choose” the desired option.

Behind this apparent simplicity though, the default effect is more complex than it seems on the surface. In fact, it has been pointed out that the underlying mechanisms can be multiple (Dinner et al., 2011) and that each default works differently based on contextual factors and on decision makers’ underlying preferences when no default is provided. In a recent metanalysis, Jachimowicz et al. (2019) have pointed out that it is necessary to reach a better grasp of the reasons behind the effectiveness of the default effect to design a more effective choice architecture (see also Zlatev et al., 2017). In other words, the default effect does not work the same way all the time as initially hypothesized. The discovery of these limitations constitutes a critique of the large proliferation of the phenomenon that has been probably both oversimplified and overgeneralized.

Theoretically, the default effect has been explained as the result of three different reasons why people may prefer not to make choices: (i) effort (ii) implied endorsement, and (iii) reference-dependent mechanisms (Johnson & Goldstein, 2003; McKenzie et al., 2006).

The first explanation implies that the default is kept because opting out would require some form of physical or cognitive effort, as for instance filling out a form or doing some calculation. We speculate that a kind of default that may be based on the aforementioned mechanism is used in website cookies disclosure. They can be all accepted as a default or personalized by removing those that are not strictly necessary. However, it is certainly more comfortable and effortless to click on the “accept all” button, especially considering the fast pace that individuals usually surf the internet.

The implied endorsement works when individuals trust in an authoritative source that has provided the preselected choice, which can be represented by a policy maker, an expert, or an advisor (Tannenbaum et al., 2017).

Finally, the reference-dependent account entails that the preselected option is coded as already chosen, becoming the status quo, thus acting like an instant endowment (Dinner et al., 2011). The endowment effect is the tendency to consider an object as more valuable when it is owned than when it is not-owned (Thaler, 1980). The logic behind it is that if we ask a certain price to sell an object we own, but we are willing to pay less for the same object as buyers, it means that the reference point is set forth by the endowment, whereas giving up the object is framed as a loss. Therefore, we ask for more money to compensate for the negative value assigned to the transaction.

In a similar way, giving up the default option can be psychologically perceived as a potential loss, thus generating reluctance in opting-out behaviors. Dinner et al. (2011) provide an alternative explanation of the psychological mechanisms responsible for this phenomenon, demonstrating that everything depends on the list of pros and cons generated in favor of the status quo. Specifically, positive aspects associated with the default and negative ones associated with alternatives (initial list) are generated before negative default and positive-alternative aspects (second list). Therefore, the initial list may result in a greater number of default pros and alternative cons compared to the last one (Query theory; Johnson et al., 2007; Weber et al., 2007).

This topic is part of a wider set of applied knowledge that has grown and developed in the last decade thanks to the diffusion of the Nudge theory (Hansen & Jespersen, 2013; Thaler & Sunstein, 2003, 2009). By definition, a nudge is *“… any aspect of the choice architecture that alters people’s behavior in a predictable way without forbidding any options or significantly changing their economic incentives”* (Thaler & Sunstein, 2008). Therefore, the default effect can be considered a nudge. Importantly, every nudge can be used in two main ways: in the interest of the choice architect, namely the subject who designs a decisional environment with the goal of prompting a certain behavioral response, or in the best interest of the decision-maker who is the target of the design process (Gigerenzer, 2015). Naturally, as we illustrated above in the example of organ donors, the benefits are intended to be possibly extended to society as a whole.

It should be a crucial aspect of choice architecture to clearly define the goal of the actions taken to shape specific choice behaviors using mechanisms that are based on decision-makers automatic, and largely unaware, mental processes.

### The joint effect of framing and defaults

Our study is devoted to investigating the default effect in a binary choice between a sure and a risky option both presented under two framing conditions: possible gains or possible losses. Moreover, to broaden our field of inquiry, we used two scenarios: a life-or-death decision and a financial decision. This setup uses a baseline in which no default is provided (no default), a condition in which the sure option is flagged (sure default), and a condition in which the risky option is flagged (risky default). Finally, we did not provide any information concerning the reason why a given option was flagged, but we asked participants to select what they believed to be the possible source of the default among four alternatives after the task. The presence of a baseline follows the recommendation of Jachimowicz et al. (2019), who pointed out the importance of comparing default effects against an estimated distribution of preferences for a specific decision problem within a given population, whereas the presence of two scenarios would help to understand how generalizable framing and defaults can be.

In this experiment, we assumed there would be three different effects exerting their influence on the evaluation of options: framing effect, scenario, and default. Although the framing effect is generally considered rather robust (see e.g., Druckman, 2001a, 2001b; Kühberger, 1998), some replications have failed to report it (Bless et al., 1998; Miller & Fagley, 1991). Nonetheless, we hypothesize that, in our experiment, a classic framing effect would influence the evaluation of the outcomes leading to risk aversion in the gain frame and risk propensity in the loss frame (H_1_).

The framing effect has been studied in relation to several possible moderators (see for instance Maule & Villejoubert, 2007; Rettinger & Hastie, 2001; Kühberger, 1998). More specifically, one study has found an interesting difference between the types of outcomes, named arena of choice, comparing possible gains/losses of either human lives or money (Fagley & Miller, 1997; hereafter we will refer to them as F&M in the text). Precisely, they have reported the main effect of the arena of choice on the overall percentage of risky choices, with a higher risk-seeking tendency with human lives than with money. Hence, we would expect to replicate a similar pattern (H_2_).

Regarding the default effect, it would presumably lead to an evaluation that favors the status quo, thus reducing the framing effect by leading individuals to be less risk seekers in the loss domain, when the default is on the sure option (H_3_), and more risk seekers in the gain domain, when the default is on the risky option (H_4_).

Finally, since our experiment leaves participants free to infer the origin of the flagged option, we will explore whether different beliefs about the default can influence its efficacy, by asking participants in the two default conditions why they think that a preselected option has been provided. This exploratory investigation would provide further information concerning the hypothesis of the implied endorsement as a potential antecedent of the default effect (Johnson & Goldstein, 2003; McKenzie et al., 2006; Tannenbaum et al., 2017).

## Materials and methods

### Participants

Nine hundred and sixty healthy volunteers (56% women; mean age 25.6 ± 7.27 S.D.) participated in the study. The sample size was calculated in line with the original study of Tversky and Kahneman (1981), using the same approximate number per cell of the design (they used approximately 155 per cell, thus we planned for 160 per cell). The sample was made up by students (70%) and other volunteers randomly recruited inside our university campus. We also involved non-students for generalizability purposes, although Druckman (2001a, 2001b) has pointed out that students do not differ from the rest of the population in the type of task used here (see also Kühberger, 1998).

### Experimental design and procedure

To investigate potential different choice patterns between decisions involving human lives and money, two scenarios were created in Italian. One was the classic ADP and the other one was an ECP purposely created for this study, which were both presented by participants. Then, three main conditions were created in two framing versions, gain and loss, resulting in six conditions administered between participants: a baseline with no default (Table 1), a sure default condition (the sure option was preselected; Table 2), and a risky default condition (the risky option was preselected; Table 3).

**Table 1:** Simulated scenarios and prospects presented in no default condition

Asian disease	Economic crisis
Imagine that the United States is preparing to face an Asian disease that, given its exceptional severity, could cause the death of 600 people. Two alternative intervention programs are proposed to deal with this event. Assume that the exact scientific estimate of the consequences of the two programs is:	Imagine that your bank is preparing to face a major financial crisis that, given its exceptional severity, could cause you to lose 60,000 € previously invested in a restricted fund in your name. Two alternative investment programs are proposed to deal with this event. Assume that the exact financial estimate of the consequences of the two programs is:
Question *“Which option would you choose?”*	
Gain frame	
*Sure prospect* If program A is adopted, 200 people will be saved *Risky prospect* If program B is adopted, there is 1/3 probability that 600 people will be saved and 2/3 probability that no one will be saved	*Sure prospect* If program A is adopted, 20,000 € will remain *Risky prospect* If program B is adopted, there is 1/3 probability that 60,000 € will remain and 2/3 probability that no money will remain
Loss frame	
*Sure prospect* If program A is adopted, 400 people will die *Risky prospect* If program B is adopted, there is 1/3 probability that no one will die and 2/3 probability that 600 people will die	*Sure prospect* If program A is adopted, 40,000 € will be lost *Risky prospect* If program B is adopted, there is 1/3 probability that no money will be lost and 2/3 probability that 60,000 € will be lost

**Table 2:** All prospects presented in sure default condition

Asian disease	Economic crisis
Question *“Would you change the preselected option in favor of the alternative?”*	
Gain frame	
*Sure prospect* If program A is adopted, 200 people will be saved *Risky prospect* If program B is adopted, there is 1/3 probability that 600 people will be saved and 2/3 probability that no one will be saved	*Sure prospect* If program A is adopted, 20,000 € will remain *Risky prospect* If program B is adopted, there is 1/3 probability that 60,000 € will remain and 2/3 probability that no money will remain
Loss frame	
*Sure prospect* If program A is adopted, 400 people will die *Risky prospect* If program B is adopted, there is 1/3 probability that no one will die and 2/3 probability that 600 people will die	*Sure prospect* If program A is adopted, 40,000 € will be lost *Risky prospect* If program B is adopted, there is 1/3 probability that no money will be lost and 2/3 probability that 60,000 € will be lost

**Table 3:** All prospects presented in the risky default condition

Asian disease	Economic crisis
Question *“Would you change the preselected option in favor of the alternative?”*	
Gain frame	
*Sure prospect* If program A is adopted, 200 people will be saved *Risky prospect* If program B is adopted, there is 1/3 probability that 600 people will be saved and 2/3 probability that no one will be saved	*Sure prospect* If program A is adopted, 20,000 € will remain *Risky prospect* If program B is adopted, there is 1/3 probability that 60,000 € will remain and 2/3 probability that no money will remain
Loss frame	
*Sure prospect* If program A is adopted, 400 people will die *Risky prospect* If program B is adopted, there is 1/3 probability that no one will die and 2/3 probability that 600 people will die	*Sure prospect* If program A is adopted, 40,000 € will be lost *Risky prospect* If program B is adopted, there is 1/3 probability that no money will be lost and 2/3 probability that 60,000 € will be lost

The way in which the default conditions differed from the baseline was twofold. One way was visual, namely that the default options were signaled by the presence of flagged selection boxes. Moreover, the task was slightly different between the baseline and the default conditions. The baseline required participants to answer the following question: *“which option would you choose?*” (see Table 1). In contrast, the default conditions required participants to answer the following question: *“would you change the preselected option in favor of the alternative?”* (see Table 2)*.*

Each participant was randomly assigned to one of the six experimental conditions and provided one choice for each individual scenario. The choice alternatives were always two: a *sure prospect*, in which the outcome was certain, and *a risky prospect*, in which the outcome was probabilistic. Expected Value was equal across the two options.

The order of presentation of the two scenarios was counterbalanced between participants’ as well as the order of the sure and the risky options.

Additionally, after the task, participants in the default conditions completed a brief survey to investigate their beliefs regarding the default option. The question presented was: *“for what reason do you think that those programs have been preselected?”*. This question was asked after participants responded to both scenarios, hence it refers to the interpretation of the default in both situations.

The four possible alternatives were: the selected program (i) was the most rational based on a logical/mathematical calculation, (ii) has been chosen by some experts, (iii) has been chosen by the majority of previous responders, (iv) it was randomly selected. The sample size was calculated based on early studies on the framing effect (Tversky & Kahneman, 1981) in which each condition had about 150 observations.

## Analysis and results

### Methodological notes

For each participant, the selection of the sure option was coded as 0, whereas the selection of the risky option was coded as 1 (for a similar method see for instance: Bless et al., 1998). Therefore, the dependent variable was an index, ranging from 0 to 1, that measured the proportion of risky choices: 0 means that responders are totally risk averse; 1 means that the responders are totally risk seeker; and 0.5 means that responders are indifferent between the two options, therefore, are risk neutral. The middle point 0.5 was also taken as a threshold against which testing whether preferences reverse or shift between conditions, considering the tendency of the overall sample. If the proportion of risky choices differs between experimental conditions but does not significantly differ from 0.5, then there is a shift in preferences. Contrary, when the proportion of risky choices not only differs between conditions, but it also differs from 0.5, then there is a choice reversal (Levin et al., 1998).

This method of measurement combines two approaches: the investigation of the bidirectional effect or choice reversal and the unidirectional effect or choice shift. (Druckman, 2001a, 2001b). Finally, it is worth specifying that this approach has been used in between experiments to measure if the overall tendency of the sample is risk neutral.

### Data analysis

Data analysis was conducted through Statistica 8.0 (StatSoft, Inc., Tulsa, OK). No missing values were detected, and all participants were included in the final analysis. The main analysis performed was a mixed repeated measure analysis of variance (ANOVA), with Scenario (ADP, ECP) as within participants’ factor, Frame (gain, loss) and Default (no default, sure default, risky default) as between participants’ factors. Additionally, we carried out an additional ANOVA adding Gender (male, female) as between factors to control for its possible moderation effects, which have been reported by F&M. An additional analysis was carried out to control for a possible correlation between the two scenarios. The significance level was set at 0.05 for all analyses.

### Results

Results show a significant main effect of Frame (*F*_1,954_ = 111.34,* p* < 0.0001, *η*^2^_*p*_ = 0.1), with a stronger tendency towards risk propensity in the loss frame (M = 0.62) than in the gain frame (*M* = 0.36). Therefore, as predicted by the framing effect, the gain frame leads participants to be generally risk averse, whereas the loss frame reverses the pattern, leading participants to be generally-risk seeking.

**Fig. 1:**
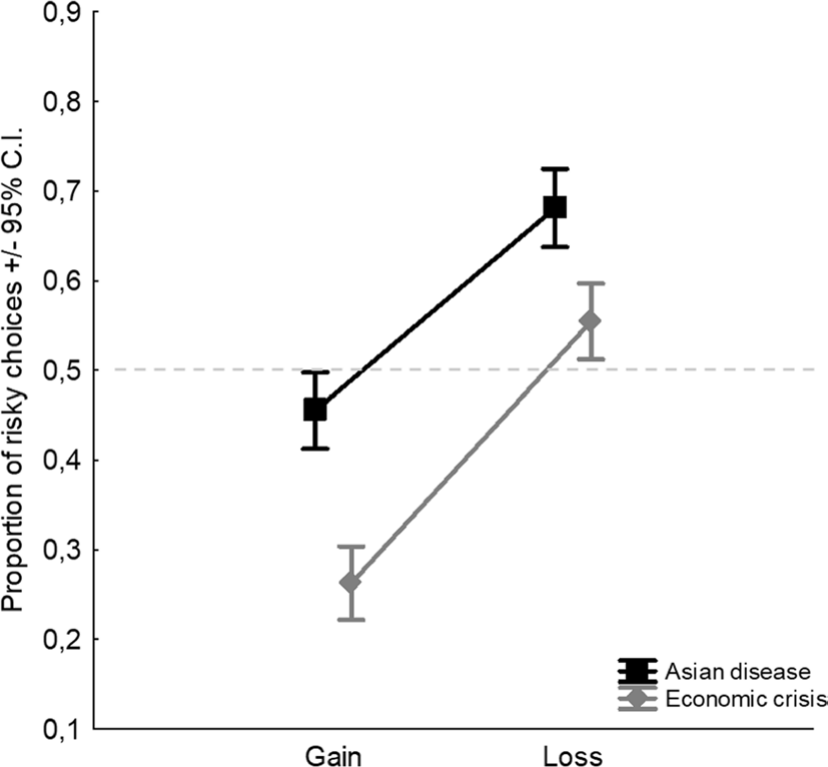
Additive effects of Frame (gain and loss) and Scenario (Asian disease and Economic crisis). Dashed line indicates the level of risk neutrality

A significant main effect of the Scenario also emerged (*F*_1,954_ = 76.15,* p* < 0.0001, *η*^2^_*p*_ = 0.07), with a higher tendency to be a risk seeker in the ADP (*M* = 0.57) than in the ECP (*M* = 0.41). Therefore, when human lives are at stake, participants are generally risk seekers, whereas when money is at stake, participants are generally risk averse. The overall arenas of choice effect that we were able to detect explained 7% of the variance of the DV. This result replicates F&M’s (1997) findings. Moreover, in line with F&M, no interaction effects between Frame and Scenario have been found. The additive (non-interactive) framing and scenario effects are reported in Fig. 1.

**Fig. 2:**
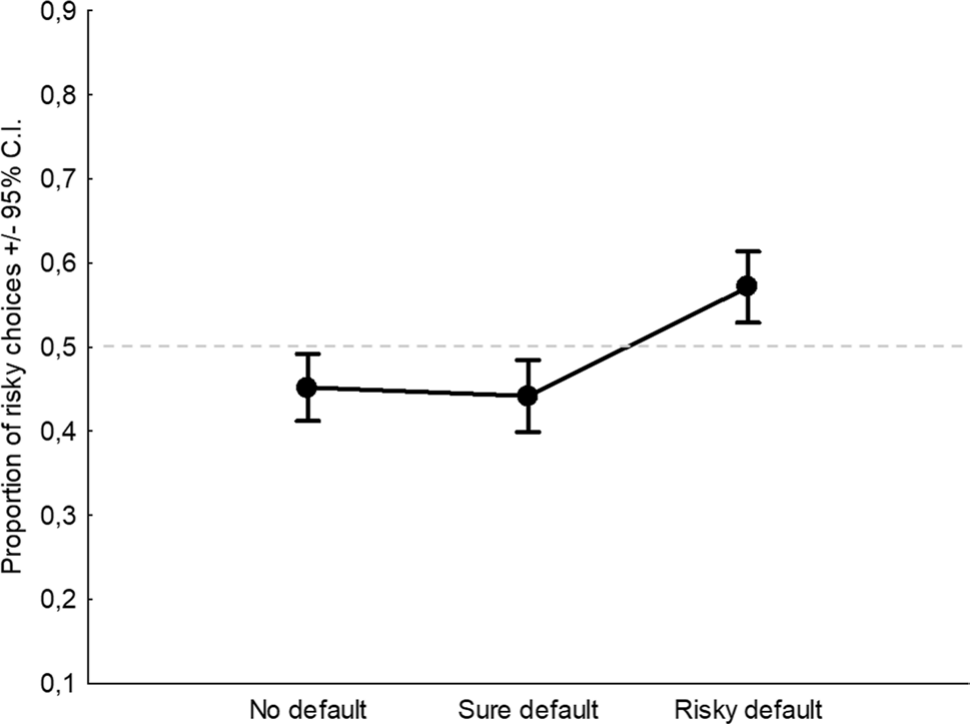
Main effect of Default. Dashed line indicates the level of risk neutrality

**Fig. 3:**
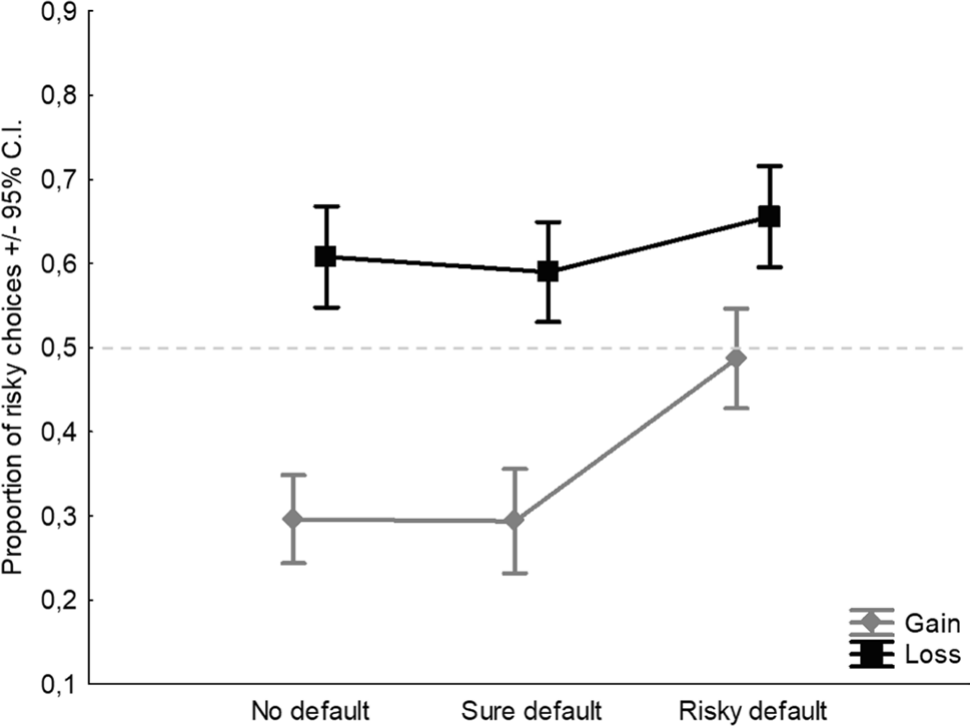
Interaction effect between Frame and Default. Dashed line indicates the level of risk neutrality

With regards to the factor Default, the main effect was found (*F*_2,954_ = 11.31,* p* < 0.0001, *η*^2^_*p*_ = 0.02; Fig. 2). Tukey post-hoc comparisons indicated that, in terms of proportion of risky choices, only the risky default condition (0.57) significantly differed from the baseline of no default (0.45), whereas the sure default condition was not different from the baseline (0.44). Overall, considering the risk neutrality threshold at 0.5, one sample *t* tests indicated that the pattern of no default (0.45) and sure default (0.44) shows risk aversion because both values fall significantly below the midline, whereas the pattern of risky default (0.57) shows risk propensity because it falls significantly above the midline (Fig. 2). All post-hoc comparisons and t-tests were significant at *p* < 0.0001. Finally, a significant but small interaction between Frame and Default was found (*F*_2,954_ = 3.4,* p* = 0.033, *η*^2^_*p*_ < 0.01; Fig. 3). Given that the interactive effect is extremely small (explaining less than 1% of the variance of the DV), we can view framing and default as essentially having independent additive effects, despite the significant interaction.

In the second analysis, Gender did not interact with any factor, thus showing no moderation effect. However, we did find an unexpected and rather small main effect of gender (*F*_2,948_ = 3.07,* p* = 0.045, *η*^2^_*p*_ < 0.01), indicating a slight tendency for women to be more risk averse than men (0.47 vs 0.52). However, although marginally significant, the effect is very small, and we consider it negligible.

The correlation between the proportion of risky choices in the two scenarios was significant (*p* < 0.001; *φ* = 0.35), indicating a moderate relationship between responses.

### Exploratory analyses and results

Additional analyses were carried out to explore the relationship between beliefs about the source of the default option and the proportion of risky choices. The majority of our participants in the two default conditions (sure and risky) believed that the default option was the most rational one based on a mathematical calculation (*n* = 314), followed by those who believed it was suggested by some experts (*n* = 151), and finally only a few participants believed that the option was selected by a majority of previous respondents (*n* = 80) or that it was randomly selected (*n* = 61). Because of the small number of the last two categories, we grouped them together into one category (*n* = 141). Therefore, we obtained a categorical variable, named *Default Source*, composed of three groups: group 1—*most rational choice*, group 2—*expert opinion*, group 3—*previous respondents/r*and*om selection*. Each group was tested against the baseline (no default) through three individual ANOVAs with Scenario (ADP, ECP) as within participants’ factor, Frame (gain, loss) and Default (no default, sure default, risky default) as between participants’ factors.

We reported the frequencies of *Default sources* across all default conditions in *Supplementary Table S1*. A chi-square test of independence was performed to examine the relation between the four experimental conditions defined by frame and default (gain sure, loss sure, gain risk, loss risk) and the frequency of respondents in each default source group. The relation between these variables was significant, *χ*^2^ (6) = 14.99, *p* = 0.02, suggesting that the assignment to a specific experimental condition was associated with the interpretation that the participant provided about the source of the default (see *Supplementary Table S1* for details on frequencies).

**Fig. 4:**
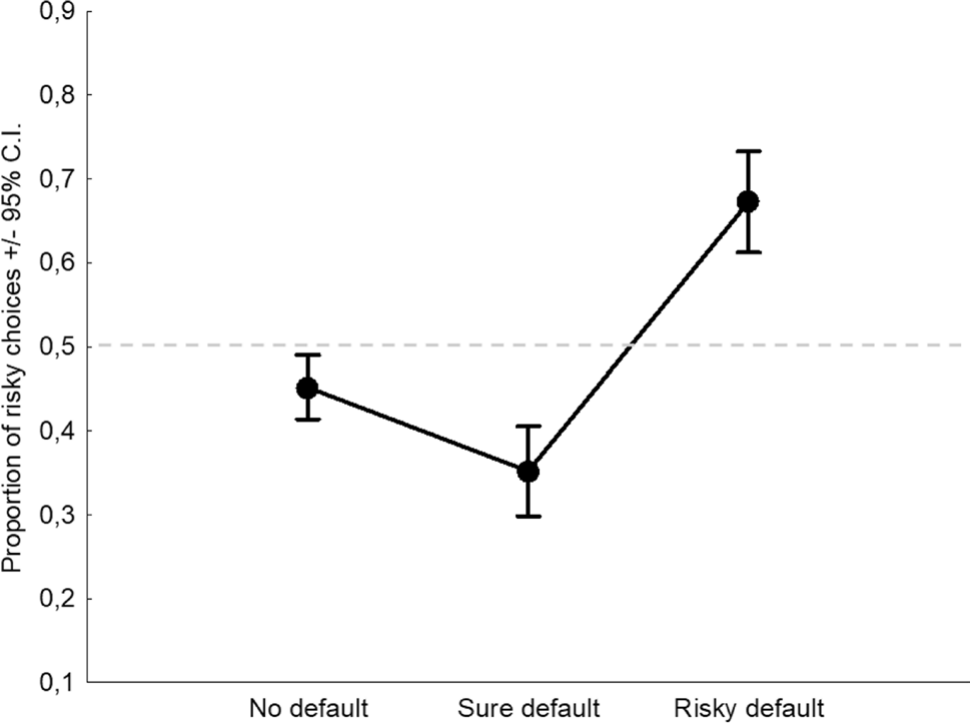
Main effect of the Default considering the groups who believed that the default, either sure or risky, was *the most rational choice based on a logical/mathematical calculation.* Dashed line indicates the level of risk neutrality

**Fig. 5:**
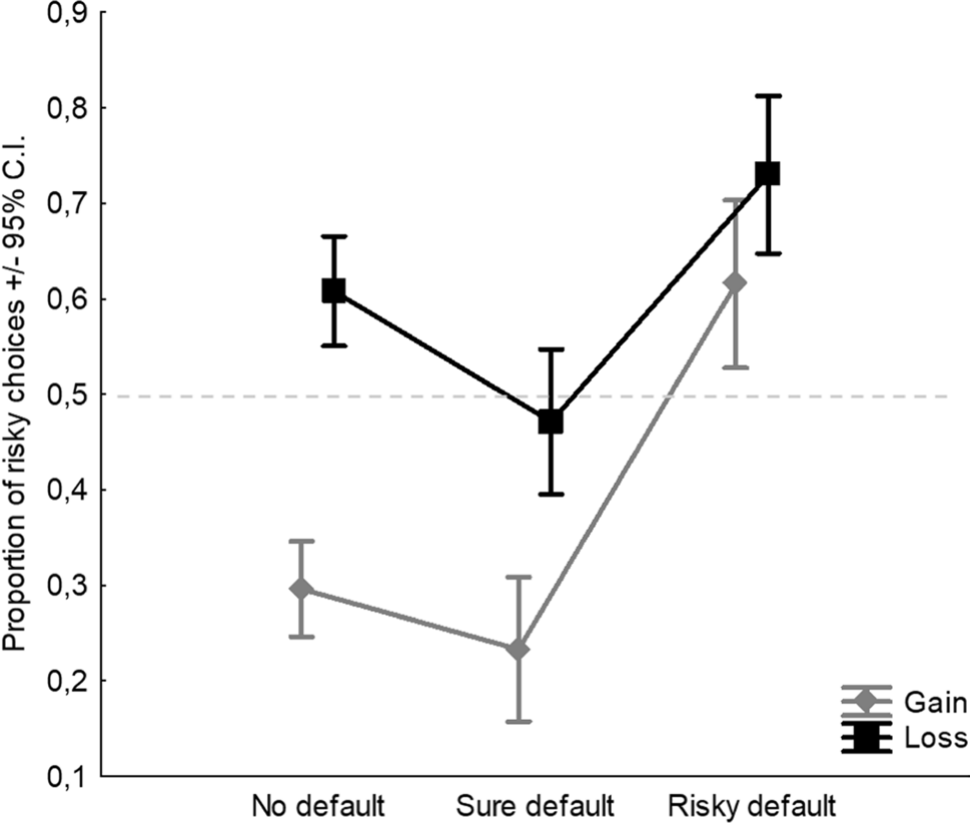
Interaction effect between Default and Frame considering the groups who believed that the default, either sure or risky, was *the most rational choice based on a logical/mathematical calculation.* Dashed line indicates the level of risk neutrality

**Fig. 6:**
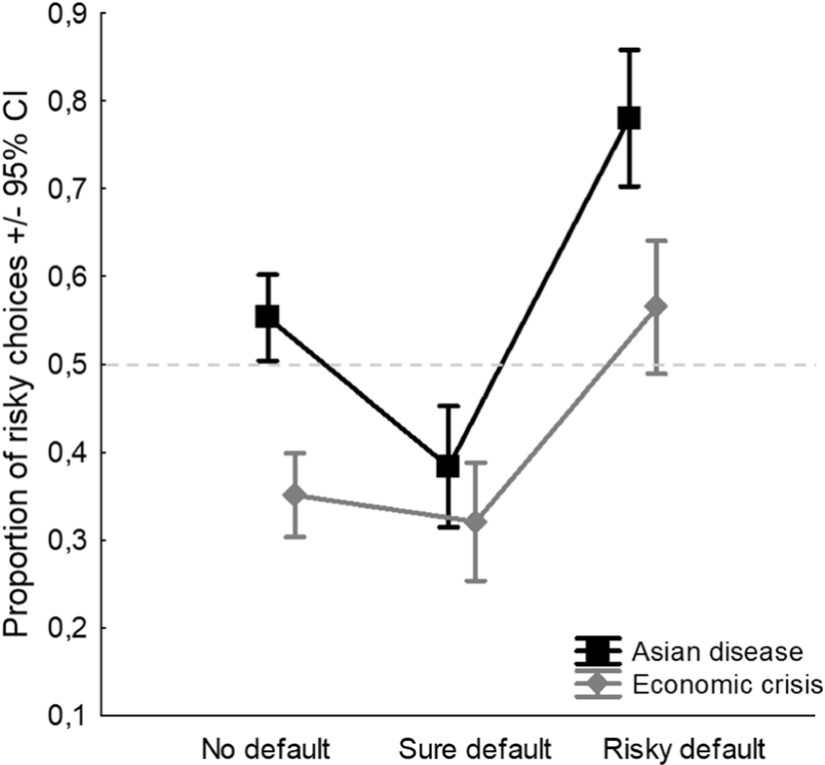
Interaction effect between Default and Scenario considering the groups who believed that the default, either sure or risky, was *the most rational choice based on a logical/mathematical calculation.* Dashed line indicates the level of risk neutrality

In group 1—*most rational choice*, the following significant effects of interest were found: a main effect of the Default (*F*_2,662_ = 31.560,* p* < 0.001, *η*^2^_*p*_ = 0.09; Fig. 4), an interaction between Default and Frame (*F*_2,662_ = 3.741,* p* = 0.024, *η*^2^_*p*_ = 0.01; Fig. 5), and an interaction between Default and Scenario (*F*_2,662_ = 4.078,* p* = 0.017, *η*^2^_*p*_ = 0.01; Fig. 6).

In group 2—*expert opinion*, the following effects of interest were not significant: main effect of the Default (*F*_2,499_ = 2.61,* p* = 0.074, *η*^2^_*p*_ = 0.01), interaction between Default and Frame (*F*_2,499_ = 2.906,* p* = 0.056, *η*^2^_*p*_ = 0.01), interaction between Default and Scenario (*F*_2,499_ = 0.295,* p* = 0.74, *η*^2^_*p*_ = 0.001).

**Fig. 7:**
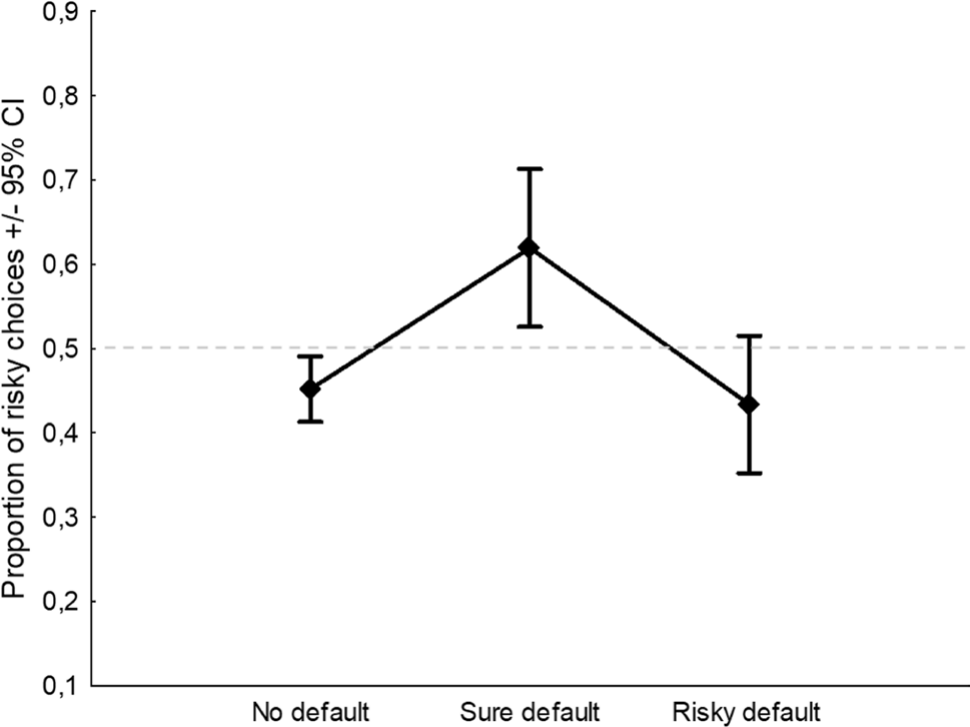
Main effect of the Default considering the groups who believed that the default, either sure or risky, was *chosen by previous respondents or r*and*omly selected.* Dashed line indicates the level of risk neutrality

In group 3—*previous respondents/r*and*om selection,* only the main effect of Default was significant (*F*_2,489_ = 5.73,* p* = 0.003, *η*^2^_*p*_ = 0.02; Fig. 7), whereas interactions were not significant: Default by Frame (*F*_2,489_ = 0.31,* p* = 0.74, *η*^2^_*p*_ = 0.001); Default by Scenario (*F*_2,489_ = 1.17,* p* = 0.31, *η*^2^_*p*_ = 0.004).

## Discussion

Our study aims to fill the gap between two streams of literature, respectively, on framing and default effects under risk and uncertainty. Taken separately, the two phenomena have had a huge impact both from theoretical and applied perspectives. However, it is worth noting that, differently from the framing effect, the mechanisms behind the default effect are still far from being properly understood. Moreover, the link between default choices and decision-making under risk and uncertainty seems to have been largely neglected. We begin with the framing effect to lay the foundation of our investigation on a solid and reliable phenomenon that provides us with useful information concerning how people form their judgements under risk and uncertainty.

### Framing effect and arena of choice

In line with our first hypothesis (H_1_), a framing effect occurred when both human lives and individuals’ own money is at stake. The consistency across the two arenas of choice found here suggests that the effect is robust against manipulation of the decision context, and it is not moderated by gender effects. Therefore, this result does not fully confirm F&M’s study that only found a framing effect limited to women. Conversely, our results are in line with Druckman (2001a, 2001b) who reported an overall large framing effect and no gender differences.

Nevertheless, our results are consistent with F&M in two respects: human lives elicit a larger tendency to risk propensity than money, confirming H_2_, and this effect does not interact with the framing. Following F&M’s reasoning, PT would have predicted an interaction between the two scenarios, because if in ECP, the gain frame polarizes the choices towards risk aversion more than ADP, it should also polarize them towards risk propensity in the loss frame. This would result in an interaction between the ADP (less polarized) and ECP (more polarized). Hence, to explain this lack of interaction, F&M hypothesized the existence of a “qualitative difference” in the way individuals make different tradeoff decisions when either human lives or money are at stake. A similar perspective can be found in Rettinger and Hastie (2001) who pointed out that the subject matter of a decision problem influences the underlying mental representations and evaluation strategies, ultimately affecting choice behavior.

More specifically, such an additive effect of scenario and framing can be simply explained by differences in evaluation strategies between life-or-death and financial decisions, as proposed by Rettinger and Hastie (2001) who support the idea that utility principles are not the only determinant of choice. For example, we may assume that, in our specific case, risk-seeking behavior is driven by the desire to save everyone, no matter the risk, which averts the possibility of an implicit decision concerning who lives and who dies, which is related to the sure option (Fagley & Miller, 1997; Shimizu & Udagawa, 2018; Tetlock, 1992). Regarding money, it may be speculated that, in both gain and loss frames, more conservative decision-making reflected the desire to limit possible losses determined by the crisis, whereas risky choices may have prompted the concern of reducing their savings to zero. Moreover, it is worth noting that these arguments build on previous research that has demonstrated the role of context as an important determinant of the type of reasoning applied to a given problem (see for instance: Maule & Villejoubert, 2007; Rettinger & Hastie, 2001).

Finally, contrary to ADP, in the loss frame of ECP we found a shift towards risk propensity, but not a reversal of preferences. Explaining this result seems relatively difficult using PT unless we allow the S-shaped value function to adapt its slope to some qualitative aspects of the outcome. On the other hand, it seems relatively less problematic to explain this result using the Fuzzy-trace theory (Kühberger & Tanner, 2010) or the Evaluative Polarity account (Wallin et al., 2016), because the shift can be directly predicted by the difference between the options in terms of attractiveness, assuming they are linguistically and emotionally asymmetrical.

A similar view can be found in the Fuzzy-trace theory (Kühberger & Tanner, 2010), which assumes a direct comparison between options, in which, in the gain frame, the sure gain is preferred over the risky gain because the latter mentions the possibility that someone will not be saved, whereas the former does not (it only mentions that 200 people will be saved). In contrast, in the loss frame, the risky loss is preferred over the sure loss because the former mentions that someone will not die, whereas the latter does not (it only mentions that 400 people will die).

In contrast, in PT the shift is predicted by the reversal of the value function, under the assumption that the options are logically equivalent, thus symmetrical, which is formally correct. However, this logic may be incompatible with the way our cognitive system processes the available information (Kühberger & Tanner, 2010). For instance, when the sure option is fully described, mentioning for instance both lives saved and not saved, the framing effect tends to disappear (Kϋhberger, 1995; Mandel, 2001), thus challenging the idea that prospects described in the form of ADP are equivalent in terms of interpretation from individuals’ perspective. This is corroborated by evidence that illustrates how people tend to make inferences concerning some part of the information that is not explicitly stated, such as the fact that “200 people will be saved” may be interpreted as “at least 200” in the gain frame and “400 people will die” may be interpreted as “at least 400” (Mandel, 2014).

### Framing and default

A first general observation is that framing and default seem to be independent additive effects. This is interesting since it suggests that they may rely on different mechanisms or mental processes, as suggested by the Additive Factor Method (Mapelli et al., 2003; Sternberg, 1969). In fact, we believe that the default effects found here are neither reference dependent nor effort dependent, simply because if that were the case, we would have observed a default effect on both sure and risky options. Nonetheless, our data suggest that default effects may be more differentiated in terms of underlying mechanisms than originally hypothesized. Importantly, an instance of a non-interactive effect between framing and default can be found in Johnson et al. (2002), although they did not compare sure and risky prospects. Therefore, compared to Johnson et al. (2002), our main advancement is that we have shown how framing and default combine their effects in decision-making under risk, which allowed us to uncover possible differences in the evaluation of sure and risky default options.

Connected to this point, another observation is that the default on the risky option, rather than on the sure one, elicited the greatest effect on a final choice. This would probably not be observed if the prospects were symmetrically represented at a cognitive level.

Regarding our hypotheses, they were only partially confirmed, although, as we will argue later, some effects were hidden by some differences in the interpretation of the default. Specifically, compared to the baseline, we did not find a significant enhancement of risk aversion due to the introduction of a default option corresponding to the sure prospect. This null result would be sufficient to reject H_3_, and to claim that the framing effect seems to elicit a stronger influence on respondents compared to the default effect. In contrast, H_4_ is clearly confirmed by the enhancement of risk propensity due to the introduction of a default option corresponding to the risky prospect. This pattern shows that the default is not inherently ineffective compared to framing, but rather that there must be something at the level of prospects evaluation that makes the sure default ineffective.

Our proposed explanation is that the risky option is felt intrinsically more attractive than the sure one because it contains the possibility of a better payoff, but its attractiveness is reduced by the tendency to overweight sure events compared to probable ones. In PT, this phenomenon is known as the certainty effect (Kahneman & Tversky, 1979). In a sense, the default may provide an external justification to be risk seekers. Although this interpretation would require further verification, it is an interesting working hypothesis that seems corroborated by a single previous preliminary study (Costa-Gomes & Gerasimou, 2020). In fact, the authors have found that the riskiest among three lotteries is preferred when proposed as the default when participants were asked whether they would like to change it. However, they did not test their hypothesis by incorporating the framing effect.

### Beliefs about the source of the default

After the riskiness of the prospects, the second important factor that influenced our default effect was the type of belief on its source, which can be viewed as an endorsement effect. Within the theories of default effects, our results would support the hypotheses of the implied endorsement mentioned in the introduction (Johnson & Goldstein, 2003; McKenzie et al., 2006; Tannenbaum et al., 2017), as a potential mechanism underlying the type of default effect found here. In this respect, our results are also consistent with Jachimowicz et al. (2019), who, in their metanalysis, have found that both endorsement and endowment reliably predict the effectiveness of the default effect. Endorsement effects, when the options were presented as credible advice from people’s own or opposing political party, have also been found in Druckman (2001b). However, it is worth mentioning that our manipulation was rather subtle, not invasive nor persuasive in any respect, and neither did it require any effort to be changed. The effect of our default was only related to the riskiness of the prospects and to what individuals attributed as the reason why one option was preselected.

In our exploratory analysis, we have found that some individuals followed the default more than others and that there seems to be a pattern that can pinpoint the link between their choice behavior and what they believe about the source of the preselected option. Let us discuss the single groups’ behavior in more detail.

In Group 1—*most rational choice*, the sure default fostered risk aversion as hypothesized in H_3_. The interaction between framing and default shows a significant shift in preferences due to the sure default in the loss condition, which leads participants to become risk neutral in a condition where they would normally be risk seekers. Moreover, the interaction between default and scenario uncovers another interesting pattern. It seems that whereas the risky default influences both ADP and ECP, the sure default only influences the ADP. It is worth recalling that the two scenarios were administered within participants, meaning that even when a person believed that the default represented the right choice in a life-or-death decision, this was not necessarily true in a financial context, and vice versa. However, considering the whole pattern mentioned above, Group 1 showed the highest level of trust in the default option.

Group 2—*expert opinion* seemed to be totally indifferent to the default, showing a choice pattern that it is only influenced by the framing and the scenario. In other words, when the default was in line with those effects, it had a greater likelihood to be accepted, whereas when it was contrary to those effects, it had a greater likelihood to be rejected. This is interesting since it suggests that individuals would potentially put more trust in the mathematical calculation of an algorithm (Group 1) than in the expertise of a human (Group 2). From these results, it can also be inferred that an expert is seen as someone who does not apply rational decision-making. This behavior may be in line with a general reluctance that sometimes individuals show regarding expert advice (Pietroni et al., 2021). Considering the whole patten mentioned above, this group showed a moderate level of distrust in the default option, which was often rejected in favor of the alternative.

Group 3—*previous respondents/r*and*om selection* rejected the default more often than the other two groups, especially in the sure default condition, where the pattern of choice reverses becoming risk seeker. Considering the whole patten mentioned above, this group showed a high level of distrust in the default option, at the point where the rejection effect worked against the tendency to be risk averse observed in the baseline when the default was in fact suggesting following that tendency. Although this group was somewhat arbitrary, because it contained two types of beliefs, the effect is interesting. The fact that chance had little endorsement is self-explanatory, but the fact that the “majority” was not trusted in a more robust fashion is more intriguing. It suggests that the simple social heuristic *imitate the majority* (Boyd & Richerson, 2005) backfires in this context. In fact, Hertwig and Hoffrage (2013) argued that even though social cues can be useful benchmarks in unknown situations, following them is not always adaptive, as it is known that the majority can make mistakes. Moreover, participants exhibited psychological reactance in this condition (Bruns et al., 2018), which is the tendency to reestablish their freedom to choose independently.

From these results, it seems clear that the default worked best in combination with the risky option, whereas the sure default only worked when two specific circumstances were converging. Specifically, when participants believed that the default was the most rational choice (Group 1), and the decision concerned human lives. In all the other circumstances, the sure default had either no effect or it led to a negative effect. This is the reason why the influence of the sure default does not emerge from the main effect illustrated in Fig. 2, whereas the effect of the risky default does emerge, although attenuated by individual differences in the beliefs about the source of the default.

However, it is worth noting that since the answer concerning the possible default source was provided after the choice made in the main task, it is possible that the judgment concerning the reason why the default was provided was actually a post-hoc rationalization, namely a way to justify their choice. This interpretation implies that, for instance, participants who were more likely to follow the default also felt more comfortable with the idea that the predetermined choice was the most rational, rather than admitting that they were influenced by expert advice, other respondents, or randomness.

Moreover, as some recent literature highlights, together with the effort people incur to select a non-default alternative, referred to as “mechanical costs” of opt-out, investigation of the default effect should also consider another relevant factor affecting opt-out, which is information or, said in other words, the knowledge people must acquire to make informed opt-out decisions (Bar-Gill & Ben-Shahar, 2021). Following this reasoning, it is possible that our participants’ tendency to differently interpret the default source has impacted their promptness toward information seeking. On the other hand, participants’ individual differences, such as for example need for cognition (e.g., Ingendahl et al., 2021), may have themselves impacted the interpretation of the default source, thus affecting the subsequent choice.

## Limitations and future research

Although insightful, our research has some limitations that are worth mentioning, along with some proposals concerning how to overcome them in future research. The main limitation concerns the way we tested the beliefs about the default. Being a mere exploratory inquiry, our goal was simply to collect some additional information concerning the phenomenon under investigation. We asked a post-hoc question (“for what reason do you think that those programs have been preselected?”) to assess a mental process that took place at the beginning of the task, during the evaluation of the decision problem. This creates a problematic interpretation of causality. In other words, we do not know to what extent participants’ choices were influenced by their beliefs about the default or if their beliefs have been influenced by the choice they made. However, the chi-square test reported above indicates that participants’ interpretation of the source of the default was partially influenced by the experimental condition they were assigned to.

More generally, at this point of our research, we are not able to assess to what extent different beliefs about the default represent the personality or other interindividual differences, or if they can be modified through contextual factors, such as different framing and default conditions, or providing information that may boost some beliefs and discourage others. Future research should be devoted to manipulating default beliefs experimentally in different scenarios, while assessing personality and interindividual differences, such as the need for cognition.

Another possible limitation is that our research has not assessed personality and interindividual differences, which could have influenced our results. For instance, some recent studies in applied psychology suggest that individuals’ risk propensity could be moderated by personality traits (see for instance Meshi et al., 2020; Aren & Hamamci, 2020) and other interindividual differences, such as the level of knowledge and experience (Mrkva et al., 2020). Hence, future research should explore the influence of interindividual differences in risk propensity in more detail.

Finally, our scenarios differed in the level of personal involvement, since in our cover story, the human lives were from another country, whereas the money was from personal savings. This could explain the differences in risk propensity. Considering this limitation, future studies should match the personal involvement between scenarios to test whether differences in risk propensity can actually be attributable to the type of outcome.

## Conclusions and practical implications

In synthesis, our results provide an interesting insight from a choice architecture perspective: it seems that, in some specific circumstances, people may not be easily nudged towards certainty, but they may be nudged towards risk. This implies the fact that special attention should be devoted to this specific feature of defaults.

More specifically, our research demonstrated that a careful analysis of the decision environment should consider multiple factors, such as: (i) the type of a decision, (ii) the current framing, (iii) the current default, (iv) the riskiness of the current default, and finally (v) its source. Based on this analysis, it may be necessary to adjust some features of the decision environment to build an effective nudging strategy or to contrast a potentially harmful one. For instance, sometimes a decision problem may be naturally framed in terms of gains, and there might be a risky default associated with it. In this case, to discourage risk propensity, the choice architect could apply two alternative strategies: remove the preselected option or setting a sure default, knowing that the latter strategy could backfire if the source of the default is misinterpreted. Some general examples of natural framings and defaults are reported below.

When people are ill, the possibility to recover is naturally framed in positive terms. This may imply a choice between alternative treatments, which are attempts to improve the current situation. When people are in perfect health, the possibility to become ill is naturally framed in negative terms. This may imply a choice between alternative programs of prevention, which are attempts to avoid the worsening of the current situation. For instance, Rothman et al. (1999) found that when the potential benefits of disease detection (such as screening for cancer) are framed in terms of losses, thus emphasizing the negative consequences of *not choosing* detection behaviors, people become more willing to follow the recommendations, to avoid possible negative consequences. This is somewhat counterintuitive compared to the natural tendency to associate early detection with its positive consequences. Therefore, the above example may be seen as a situation in which the no-detection behavior is the natural default and reframing it in loss terms could help people focus on the potentially negative consequences of inaction.

In the financial domain, investments may be naturally framed in positive terms, whereas financial problems may naturally be framed in negative terms. In these circumstances, whenever people have different options, one of them could be the preselected choice. It could be an investment habit, a flag on a form, or a default strategy that the bank proposes to its customers. For instance, a strategy that aims to maximize returns would be a risky default option, whereas one that aims to minimize risks would be a conservative default option.

To mention one last domain briefly noted in the introduction, it seems that default settings on website cookies exploit both a default and a loss framing. When cookies acceptance is set by default, users can opt-out, but they are often warned that some functions of the website may not work if they do so, thus empathizing the negative consequences of not choosing the default.

Finally, it is worth recalling what we stated in the introduction regarding possible nudges or behavioral strategies in general. From an ethical perspective, it is always crucial to define who will benefit from specific interventions before approving their application.

## Supplementary Information

Below is the link to the electronic supplementary material.

Supplementary file1 (DOCX 17 KB)
