# Supplementary material for: The joint effect of framing and defaults on choice behavior
Source: Psychol Res. 2022 Sep 5;87(4):1114–28. doi: 10.1007/s00426-022-01726-3 (PMC10192178; doi:10.1007/s00426-022-01726-3)
Supplement: Supplementary file 1 — Supplementary file1 (DOCX 17 KB) [file 426_2022_1726_MOESM1_ESM.docx]

**The joint effect of framing and defaults on choice behavior**

Felice Giuliani^1, 2^, Loreta Cannito^1,3^, Gilberto Gigliotti^2^, Angelo Rosa^4^, Davide Pietroni^2^ & Riccardo Palumbo^1, 2^

^1^ Center for Advanced Studies and Technology, University “G. d’ Annunzio” of Chieti-Pescara, Chieti, Italy

^2^ Department of Neuroscience, Imaging and Clinical Sciences, University “G. d’ Annunzio” of Chieti-Pescara, Chieti, Italy

^3^ Department of Psychological Sciences, Health and Territory, University “G. D’Annunzio” of Chieti-Pescara, 66100, Chieti, Italy

^4^ Department of Management, Finance and Technology, University “LUM", Casamassima, Bari, Italy

**Supplementary material**

|  | | | **Default Source** | | | Total |
| --- | --- | --- | --- | --- | --- | --- |
|  |  |  | Most  Rational Choice | Expert Opinion | Previous Respondents/Random  Selection |  |
| **Experimental condition** | Gain Sure | Frequency | 88 | 26 | 29 | 143 |
|  |  | Expected Frequency | 74.1 | 35.6 | 33.3 | 143.0 |
|  |  | Residual | 13.9 | -9.6 | -4.3 |  |
|  | Loss Sure | Frequency | 87 | 38 | 31 | 156 |
|  |  | Expected Frequency | 80.8 | 38.9 | 36.3 | 156.0 |
|  |  | Residual | 6.2 | -.9 | -5.3 |  |
|  | Gain Risk | Frequency | 65 | 44 | 47 | 156 |
|  |  | Expected Frequency | 80.8 | 38.9 | 36.3 | 156.0 |
|  |  | Residual | -15.8 | 5.1 | 10.7 |  |
|  | Loss Risk | Frequency | 74 | 43 | 34 | 151 |
|  |  | Expected Frequency | 78.2 | 37.6 | 35.1 | 151.0 |
|  |  | Residual | -4.2 | 5.4 | -1.1 |  |
| Total | | Frequency | 314 | 151 | 141 | 606 |
|  |  | Expected Frequency | 314.0 | 151.0 | 141.0 | 606.0 |

Table S1 – Frequency of respondents in each of the three default source groups across the four experimental conditions.
